# Prevalence and Risk Factors of Restless Legs Syndrome among Chinese Adults in a Rural Community of Shanghai in China

**DOI:** 10.1371/journal.pone.0121215

**Published:** 2015-03-24

**Authors:** Yunbo Shi, Huan Yu, Ding Ding, Peimin Yu, Dongyan Wu, Zhen Hong

**Affiliations:** Department of Neurology, Huashan Hospital, Fudan University, Shanghai, China; Catholic University of Sacred Heart of Rome, ITALY

## Abstract

**Aims:**

The primary objective of this study was to investigate the prevalence and risk factors of restless legs syndrome (RLS) in an adult Chinese population living in a rural community. We also aimed to determine the predictive diagnostic value of the 4-item screening questionnaire for RLS in this population.

**Methods:**

This study was designed as a 2-phase survey. In phase 1 we performed a face-to-face interview of eligible individuals living in a rural community in Shanghai using a 4-item screening questionnaire. In phase 2, sleep specialists performed a phone interview of the individuals who screened positive to diagnosis RLS.

**Results:**

Forty-one RLS cases were confirmed among 2941 eligible individuals 18 years of age or older in the study community. The prevalence of RLS was 1.4% (95% confidence interval (CI) =1.0-1.9%), with a significantly higher rate observed in females (1.9% [95%CI =1.3-2.7%]) than that in males (0.9% [95%CI =0.5-1.5%], p=0.019). The prevalence rate increased significantly with age, from 0.2% (95% CI =0.08-0.6%) in those 18-39 years old to 4.1% (95% CI =2.1-7.9%) in those ≥70 years old (p<0.001). The multivariate logistic regression analysis indicated that gastritis, anemia and hypertension were risk factors for RLS. The sensitivity and specificity of the 4-item screening questionnaire used in this study were 63.4% and 97.5%, respectively.

**Conclusion:**

RLS prevalence is relatively low among Chinese adults living in rural Shanghai. Furthermore, population-based studies with a larger sample size and a longitudinal follow-up may help to determine the risk factors of RLS and potential interventions for RLS.

## Introduction

Restless legs syndrome (RLS) is a neurological sensorimotor disturbance characterized by distressing sensations in the limbs, particularly the legs, that begin or worsen during periods of inactivity. The sensations are worse during the evening and nighttime hours and are partially or totally relieved by movement [1,2]. More recently, research has shown that RLS negatively affects sleep [3] and quality of life [4] and increases the risk of coronary heart disease [5]; thus, RLS is drawing the attention of an increasing number of neurologists.

More epidemiological studies have been conducted since the International Study Group declared the diagnostic criteria for RLS (1,2). The prevalence of RLS in residents of Western countries >18 years of age was estimated to be 8.5%-28.2% [6,7]. The prevalence of RLS in Asian populations has been shown to be much lower, ranging from 0.1% to 12% [8,9]. This difference is thought to be due to genetic heterogeneity and the geographic locations and differences in research methodology of these studies [10].

Previous studies have shown that translating the diagnostic criteria of RLS into different languages can result in wording variations due to the idiosyncrasies of each language. Also, the study procedure, or social and cultural factors can cause significant difficulties when diagnosing RLS. Based on these considerations, it was strongly recommended that the sensitivity, specificity, and positive predictive value of any instrument used to diagnose RLS should be validated for the population in which it is intended to be used [11]. To our knowledge, only two RLS studies were previously conducted in a Chinese population in mainland China [10,12]. However, neither of those studies validated the instruments that they used in their study population.

In this population-based epidemiological study, we aimed to investigate the prevalence and risk factors of RLS in Chinese adults ≥18 years of age living in a rural community in Shanghai, China. We also validated the 4-item screening questionnaire for RLS in this population.

## Methods

### Ethics Statement

This study was approved by the Medical Ethics Committee of Huashan Hospital, Fudan University, Shanghai, China. A written informed consent was obtained from all of the participants and/or their legal guardian.

### Study population

The present door-to-door survey was conducted in a community of Shanyang town, Jinshan district, Shanghai, China, from January, 2013 to February, 2013. Potential subjects were identified using a government maintained ‘residents list’, which includes the name, sex, age, address, and telephone number of every resident. To be included in the study, the participant was required 1) to be a registered resident in the community; 2) to be ≥18 years of age; and 3) to provide a written informed consent for study participation.

### Screening procedure of the study

At phase I of the study, a screening questionnaire was used by the physicians and nurses to identify potential RLS cases. Demographic information was collected for each study subject (e.g., age, sex and the number of pregnancies in females). We also recorded the participants’ self-reported height and weight (overweight was defined as BMI>25 kg/m^2^) and life style factors (e.g., smoking and drinking: smoking was defined as smoking at least one cigarette per day in the year prior to the study and regular drinking was defined as consuming at least 250 ml of beer or 50 gm of Chinese alcohol more than twice a week). Medical histories, such as physician-diagnosed hypertension, diabetes, anemia, gastritis, kidney disease, cardiovascular disease, and mental disorders (depression and anxiety) were also collected. Four questions suggested by the International Restless Legs Syndrome Study Group (IRLSSG) [2] based on the symptoms of RLS were used for screening. These questions were as follows: (1). Do you have unpleasant sensations (for example, crawling, tingling, pulling, stinging, burning, etc.) in your legs combined with an urge or need to move your legs? (2). Do these feelings occur mainly or only when you are at rest or lying down? (3). Do these feelings improve with movement (for example, walking, stretching, flexing, rubbing your legs or other activities)? (4). Are these feelings worse in the evening or night than in the morning? A sleep specialist at Huashan Hospital translated these four questions into Chinese.

### Diagnosis of RLS

Those subjects who answered “yes” to question 1 alone or to question 1 and any additional questions during phase I of the study were identified as potential RLS cases at phase II. Sleep specialists at Huashan Hospital interviewed the participants over the telephone to rule out conditions mimicking RLS, such as radiculopathy and arthritis, etc. A RLS patient was defined as someone whose symptoms were consistent with RLS diagnostic criteria, with no possibility of having a disease that could mimic RLS. The degree of RLS severity was determined according to the 10-item IRLSSG rating scale: mild, (IRLS scores, 1–10); moderate, (IRLS scores, 11–20); severe, (IRLS scores, 21–30); and very severe,(IRLS scores, 31–40))[13]. The participants’ history of RLS-like symptoms among close relatives and the onset age of RLS symptoms were also recorded.

### Statistical analysis

The categorical variables were expressed as frequencies (%). The Pearson Chi-squared test and Cochran Mantel Haenszel Chi-squared test were used to compare the categorical variables. The prevalence and 95% confidence intervals (CIs) of RLS were calculated for the entire population and for the population by age and gender. A multivariate logistic regression analysis was used to detect the risk factors associated with RLS in the total population and in males and females. The models were adjusted for demographic factors, life styles, and medical histories. Factors with a frequency <10% were not entered into the regression model.

All of the p-values and CIs were estimated in a two-tailed manner. Differences were considered to be statistically significant at p<0.05. The data were analyzed using SPSS 17.0.

## Results

### Characteristics of the study subjects

**Fig 1:**
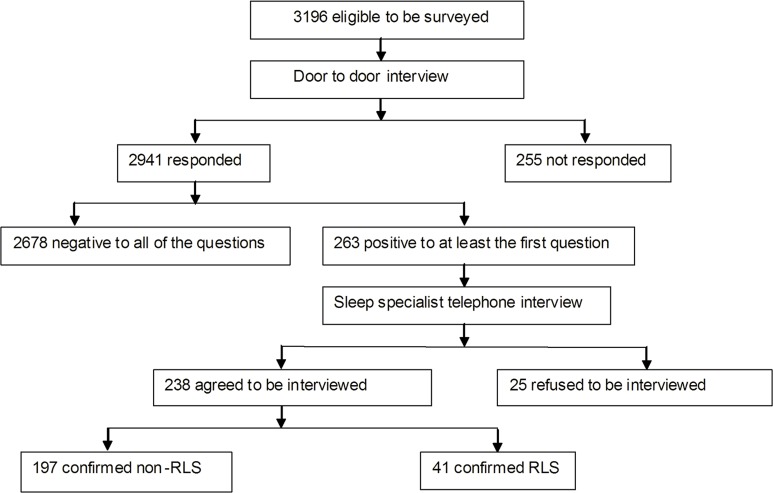
Flow chart of patients with RLS through the study. 3196 subjects were enrolled in this study. 2941 subjects completed phase I screening using 4-item questionnaire to identify potential RLS cases. 263 subjects who at least answered “yes” to question 1 during phase I of the study were identified as potential RLS cases at phase II. At last, 41 subjects were diagnosed RLS patient consistent with RLS diagnostic criteria, with no possibility of having a disease that could mimic RLS by Sleep specialists.

Fig 1 shows that, 3196 registered residents who met the inclusion criteria were contacted from 2 neighborhoods in the community. Among these subjects, 255 (7.98%) refused to participate. Thus, 2941 subjects completed phase I screening, and 263 subjects among them entered phase II of the study as potential RLS cases. During phase II, the sleep specialist interviewed 238 potential RLS cases (25 refused to be interviewed) and diagnosed 41 participants with RLS.

### RLS prevalence

The RLS prevalence in our sample was 1.4% (95% CI: 1.0–1.9%). The RLS prevalence in females (1.9%, 95% CI: 1.3–2.7%) was twice as high as that in males (0.9%, 95% CI:0.5–1.5%, p = 0.019). RLS prevalence increased significantly with age. The RLS prevalence in the 18–39-year-old age group was 0.2% (95% CI: 0.08–0.6%), while that for the ≥70-year-old age group was 4.1% (95% CI: 2.1–7.9%, p<0.001) (Table 1).

**Table 1:** Prevalence of RLS by sex and age-group.

	Study population	RLS cases, n	Prevalence, % (95%CI)	P value
Total	2941	41	1.4 (1.0–1.9)	
Sex				
Male	1467	13	0.9 (0.5–1.5)	0.019
Female	1474	28	1.9 (1.3–2.7)	
Age-group				
18–39	1375	3	0.2 (0.08–0.6)	<0.001
40–49	598	7	1.2 (0.58–2.4)	
50–59	480	13	2.7 (1.6–4.6)	
60–69	294	10	3.4 (1.9–6.1)	
≥70	194	8	4.1 (2.1–7.9)	

### Socio-demographics and medical history of people with and without RLS

Sixty-eight percent of people with RLS were female, which was significantly higher than people without RLS (50%). The average age of people with RLS was significantly higher than that of people without RLS (59.1 years (SD 16.0) vs. 43.3 years (SD 15.4)). The average BMI of people with RLS was significantly higher than that of people without RLS (24.3 (SD 4.5) vs 22.7 (SD 3.3)). However, when being overweight was defined as BMI>25 kg/m^2^, there was no significant difference in the frequency of overweight individuals with or without RLS. People with RLS had significantly less years of education than people without RLS (5.7 (SD 3.9) vs. 10.4 (SD 4.4)). There was no significant difference in regular alcohol consumption, regular smoking or heart disease between the 41 RLS cases and 2875 non-RLS cases. Compared to the non-RLS cases, people with RLS were more likely to have diabetes, mental disorders, anemia, kidney disease, gastritis, and hypertension (Table 2).

**Table 2:** Socio-demographics and medical history of individuals with or without RLS.

	RLS (n = 41)	Non-RLS (n = 2875)	P value
Female, n(%)	28 (68.3)	1434 (49.9)	0.019
No. pregnancy*	23	815	0.017
0–1	9(39.1)	532 (65.3)	
2	7(30.4)	177 (21.7)	
≥3	7(30.5)	106 (13.0)	
Age, mean (SD)	59.1 (16.0)	43.3 (15.4)	<0.001
Education duration, mean (SD)	5.7 (3.9)	10.4 (4.4)	0.001
BMI, mean (SD)	24.3±4.5	22.7±3.3	0.002
Overweight, n (%)	12(29.3)	539(19.2)	0.112
Regular alcohol drinker, n (%)	4 (9.8)	499 (17.4)	0.200
Regular smoker, n (%)	6 (14.6)	705 (24.5)	0.142
History of disease, n (%)			
Diabetes	6 (14.6)	90 (3.1)	0.002
Mental disorder	3 (7.3)	13 (0.5)	0.001
Anemia	8 (19.5)	88 (3.1)	<0.001
Kidney disease	3 (7.3)	21 (0.7)	0.004
Gastritis	9 (22)	128 (4.5)	<0.001
Hypertension	24 (58.5)	417 (14.5)	<0.001
Heart disease	3 (7.3)	84 (2.9)	0.122

*Among female subjects

### Risk factors associated with RLS

The results of the multivariate logistic regression analysis of the factors impacting RLS are presented in Table 3. The analysis indicated that gastritis (OR = 2.96; 95% CI:1.27–6.90; P = 0.012), anemia (OR = 6.93; 95% CI:2.78–17.30; P<0.001), and hypertension (OR = 4.10; 95% CI: 1.88–8.92; P<0.001) were risk factors for RLS. For the female population, gastritis (OR = 3.28; 95% CI:1.05–10.24; P = 0.041) and anemia (OR = 10.86; 95% CI:3.87–30.48; P<0.001) were risk factors of RLS, and for the male population, only hypertension (OR = 7.18; 95% CI:1.74–29.58; P = 0.006) was the risk factor of RLS.

**Table 3:** Odds ratios of factors associated with RLS in total, male and female populations, by multivariate logistic regression analysis.

	Total		Male		Female	
Variables	OR (95% CI)	P value	OR (95% CI)	P value	OR (95% CI)	P value
Sex	1.35 (0.57–3.20)	0.501	-	-	-	-
Age	1.02 (0.99–1.05)	0.209	1.04 (0.98–1.10)	0.210	1.01 (0.96–1.06)	0.845
Education year	0.91 (0.83–1.00)	0.070	0.88 (0.73–1.05)	0.164	0.93 (0.82–1.06)	0.294
Overweight	1.31 (0.63–2.73)	0.476	2.01 (0.56–7.16)	0.282	1.11 (0.37–3.34)	0.851
No. pregnancy	-	-	-	-	1.23 (0.79–1.91)	0.354
Drinking alcohol regularly	0.72 (0.21–2.53)	0.612	0.66 (0.18–2.49)	0.543	-	-
Smoking regularly	0.85 (0.27–2.64)	0.773	1.08 (0.32–3.68)	0.903	-	-
Diabetes	1.75 (0.64–4.82)	0.278	2.84 (0.53–15.14)	0.222	1.69 (0.39–7.40)	0.488
Anemia	6.93 (2.78–17.30)	<0.001	-	-	10.86 (3.87–30.45)	<0.001
Gastritis	2.96 (1.27–6.90)	0.012	3.59 (0.90–14.27)	0.070	3.28 (1.05–10.24)	0.041
Hypertension	4.10 (1.88–8.92)	<0.001	7.18 (1.74–29.58)	0.006	2.75 (0.91–8.34)	0.074
Heart disease	0.56 (0.15–2.17)	0.402	0.58 (0.07–5.21)	0.628	0.30 (0.03–3.20)	0.320

### The family history and severity of RLS cases

Among 41 diagnosed RLS cases, 7 (17.1%) reported a family history of RLS-like symptoms among relatives of any degree. Of those diagnosed with RLS, 31.7% had mild symptoms, 53.7% had moderate symptoms and 14.6% had severe symptoms. Only nine patients (21.9%) were aware of their disease and diagnosed RLS by a physician.

### Validation of the screening questionnaire

Among the 238 interviewed potential RLS cases, 31 subjects responded “yes” to all four questions, and 26 of those 31 subjects were diagnosed RLS. Also, 15 out of 207 subjects who answered “yes” to at least the first question, but not to all four questions, were diagnosed with RLS. The 4-item screening questionnaire had a 63.4% sensitivity and a 97.5% specificity for the diagnosis of RLS in this study population. Its positive predictive value was 83.9%, and its negative predictive value was 92.8%.

## Discussion

The prevalence of RLS was found to be 1.4% in the current population-based Chinese study using a door-to-door interview and telephone interview performed by sleep specialists according to the criteria of IRLS. This finding is substantially lower than the prevalence rates documented for primarily adult Caucasian population samples (7.2%–14.3%) [6,14,15], but is similar to prevalence rates documented for most adult Asian population samples (1.57%–2.1%) [16,17,18].

To date, two other studies have reported the prevalence of RLS in mainland China [10,12]. One was conducted in a population of 16 years of age and above in a town of Shandong province. The diagnosis in that study was based only on the 4-item screening questionnaire suggested by the IRLSSG, and no further interview was performed by a sleep specialist. Thus “mimics” (false positive diagnoses of RLS) could not be excluded. In that study, the prevalence of RLS was 7.2% [12], which was much higher than that in our study (1.4%, ≥18 years). Another 3-step-design study surveyed an elderly population, over 50 years of age, in a town of Shanghai. They used the 4-item screening questionnaire suggested by the IRLSSG to perform the first telephone interview. The second telephone interview was performed by a sleep specialist to rule out ‘mimics’ and secondary RLS. The final face-to-face interview was performed in the clinic for confirmation and examination. The overall prevalence of primary RLS was 0.69% in the study [10], which was lower than that in our study (3.2%, ≥50 years). A possible explanation for the differences in the results between our study and the previous studies is differences in procedure and sample selection.

Recently, it has become clear that the positive predictive value of many questionnaire screens for RLS may be fairly low, and many individuals who are identified by these screens have other conditions that can “mimic” the features of RLS by satisfying the four diagnostic criteria. The data from diagnostic studies on RLS have indicated that the use of the essential diagnostic criteria of the IRLSSG by RLS experts has less than perfect sensitivity and specificity. ‘‘Mimics” (false positive diagnoses) of RLS have been reported in 16% of the investigated populations [11]. In another study, up to 41.0% of the individuals that replied ‘yes’ to either one, two or three questions asked by the interviewers were diagnosed with RLS [19]. The gold standard for the correct diagnosis of RLS is a careful and detailed interview by an experienced specialist [20]. Hening et al. emphasized that the 4-item screening questionnaire was sensitive, specific and reliable for RLS diagnosis even if it was used during a telephone interview, but the interview should be conducted by a sleep specialist. The results of RLS diagnosis using this questionnaire would be different if the questions were asked by non-physician interviewers [21]. Based on these considerations, the 4-item screening questionnaire should be evaluated for its sensitivity, specificity, and positive predictive value for its target population. Based on the results of phase I and phase II of our study, we validated the 4-item screening questionnaire and concluded that its sensitivity and specificity were 63.4% and 97.5%, respectively. Its positive predictive value and negative predictive value were 83.9% and 92.8%, respectively. As we know, this was the first study to determine the predictive value of the 4-item screening questionnaire in a Chinese population.

Although the prevalence of RLS varies widely in different ethnic groups, many studies have found the prevalence of RLS in females to be twice as much as in males, and RLS prevalence has also been found to increase significantly with age [4,22,23]. We found similar trends in our study. Previous studies have shown that subjects suffering from anemia and gastrointestinal disorders have a significantly increased risk of RLS [24,25], and most of RLS patients have Periodic Limb Movements During Sleep(PLMS), which can cause hypertension [26]. The logistic regression analysis indicated that the risk factors of RLS in our study population were anemia, gastritis and hypertension. Using multivariate logistic regression analysis, Bjorvatn et al found that gender and age group did not show clear significant relationships with RLS [15]. In our study, after adjusting for the factors of social-demographics and medical history, gender and age were also not recognized as risk factors of RLS. Thus, although RLS is more prevalent in females, there may be other factors that can explain this reported gender difference. Interestingly, further subgroup analysis indicated that the different genders had different risk factors. Among the female population, gastritis and anemia were risk factors of RLS, while for the male population, only hypertension was a risk factor of RLS. The reasons of these different remains to be evaluated. But as we know, Gastritis and Anemia both can cause low serum ferritin. Iron status has to be considered as a primary neuropathological factor of RLS, while iron status differs markedly for different genders. The serum ferritin values of female lower than males, Juuti et al using a well validated field-dependent relaxation-rate-increase measurement showed significantly lower brain iron for adult women than men[27], The large gender differences in iron status do not suffice to increase the risk of RLS unless another factor further stresses the iron stores. When female have Gastritis and Anemia, very low serum ferritin exacerbates or even engenders RLS symptoms. That may be one of the reasons why Gastritis and Anemia were the risk factors of female RLS, but not male RLS. Mallon et al found men with RLS without daytime sleepiness were more often had hypertension compared to men in the reference group, while there was no significant difference of hypertension between women with RLS without daytime sleepiness and women in the reference group [28]. Batool-Anwar et al found that, for females, after adjusting for social-demographic factors, hypertension was an risk factor of RLS, when the symptom frequency ≥15 times/month, yet it was not the risk factor of RLS, when the symptom frequency was 5–14 times / month. They indicated that women with RLS have a higher prevalence of hypertension, and this prevalence increases with more frequent RLS[29]. In our study, social-demographics and medical histories were taken into consideration, further subgroup analysis indicated that hypertension was an RLS risk factor for males but not females. In our study, 87.5% of RLS cases were of mild-moderate severity; and the frequency of RLS in our study population was low, which can partly explain our results. Therefore, future studies with larger samples and longitudinal follow-up should examine whether there are gender differences in the risk factors of RLS.

Another important finding in our study was that only nine patients were aware of their disease and had been diagnosed with RLS by a physician. This ignorance may result from people not viewing RLS as a disease. This finding shows that RLS is under-diagnosed due to the low physician awareness of RLS in mainland China.

There were some advantages of this study. First, this door-to-door study had a high response rate of 92.0%. In addition, we are the first to validate the diagnostic ability of the 4-item screening questionnaire in a Chinese population. Furthermore, we demonstrated the risk factors for different genders. Several limitations of this study should be taken into account. First, 25 of the 263 participants that responded ‘yes’ to at least the first question refused to participate in phase II, which might have resulted in an underestimation of the prevalence of RLS; however, the refusal rate was low, so the results should be reliable. Secondly, all of the clinical disorders were self-reported, which can generate recall bias. Thirdly, our sample was from a rural community of Shanghai, where the socio-economic status is higher than in the rural communities of other cities. Therefore, the data cannot be generalized to the entire 1370 million Chinese living throughout the country. Lastly, the use of cross-sectional data prohibits any assessment of the causal relationship between RLS and the clinical disorders surveyed. The causal relationship may be verified in future follow-up studies for this cohort.

Our study results suggest that the prevalence of RLS was 1.4% among Chinese adults in a rural community of Shanghai in China, but up to 78.1% RLS patients have not been diagnosed or treated. An increasing number of studies have shown that RLS negatively affects sleep and quality of life and increases the risk of coronary heart disease and hypertension, yet medicines can efficiently treat this disease. Therefore, there is a need to increase the awareness regarding RLS among both medical professionals and the general population. Further population-based studies with larger sample sizes and longitudinal follow-up may help to determine the risk factors of RLS and potential interventions for RLS.
